# CircPDSS1 promotes the proliferation, invasion, migration, and EMT of breast cancer cell via regulating miR-320c/CKAP5 axis

**DOI:** 10.1186/s12935-022-02657-0

**Published:** 2022-07-28

**Authors:** Xia Liu, Jingyong Song, Yu Kang, Yaojia Wang, Anyue Chen

**Affiliations:** Department of Breast Oncology, Hainan Cancer Hospital, West fourth Street, Changbin, Xiuying District, Hainan, 570100 China

**Keywords:** Breast cancer, circPDSS1, miR-320c, CKAP5

## Abstract

**Background:**

Breast cancer (BC) poses serious threats to women’s health. A large number of reports have proved that circular RNAs (circRNAs) exert vital functions in human cancers, including BC.

**Methods:**

The function of circPDSS1 in BC cells was tested by CCK-8, colony formation, TUNEL, transwell-invasion, wound healing, and IF assays. RNA pull down, luciferase reporter and RIP assays were employed to verify the relationship among circPDSS1, miR-320c and CKAP5.

**Results:**

CircPDSS1 was upregulated in BC cells, and circPDSS1 knockdown repressed BC cell malignant behaviors. Further, circPDSS1 was found to bind to miR-320c in BC cells, and miR-320c overexpression suppressed malignant processes of BC cells. MiR-320c could also bind to CKAP5. Moreover, miR-320c inhibition increased the level of CKAP5, but circPDSS1 downregulation decreased the level of CKAP5. Finally, rescue experiments indicated that CKAP5 knockdown countervailed the promoting effect of miR-320c inhibition on the malignant behaviors of circPDSS1-depleted BC cells.

**Conclusions:**

CircPDSS1 promotes proliferation, invasion, migration as well as EMT of BC cells by modulating miR-320c/CKAP5 axis. Our finding may be useful for researchers to find new potential therapeutic or diagnostic targets for BC.

## Background

Breast cancer (BC) belongs to the most common invasive cancers among women and has caused lots of deaths globally [1]. Despite that plenty of treatment strategies have been developed over the past years, the occurrence of BC keeps an increasing tendency. In the meantime, BC patients usually have poor prognosis [2]. Moreover, advanced BC patients have to sustain extremely depressing prognosis due to the metastasis and chemo-resistance of cancer [3]. For the purpose of strengthening the treatment effect of varied therapies for BC, it is essential to find promising early diagnostic biomarkers and new treatment targets. Therefore, comprehending the molecular mechanism in BC cells is critically needed.

Circular RNAs (circRNAs) are identified as a cluster of non-coding RNAs possessing stable and closed loop without protein-coding ability [4]. An increasing number of researches have elucidated that ectopic expression of circRNAs is linked to the development of various cancers, including BC [5]. For instance, circRNA-000911 plays an anti-oncogenic part in BC and might work as a promising therapeutic target for BC patients [6]. A novel FLI1 exonic circRNA facilitates metastasis of BC by modulating DNMT1 and TET1 [7]. Moreover, it has been uncovered that circRNAs can function as competing endogenous RNAs (ceRNAs) to competitively bind with microRNAs (miRNAs) and modulate the expression of downstream messenger RNA (mRNAs), consequently affecting the pathological activities in BC. For instance, circRNA_002178 accelerates BC progression by upregulating miR-328-3p-mediated COL1A1 [8]. Furthermore, circ0052112 targets miR-125a-5p to facilitate the invasion and migration of BC cells [9]. CircAGFG1 functions as a sponge for miR-195-5p to boost the development of triple-negative BC via regulating the expression of CCNE1 [10]. As for the cancer-promoting role of circPDSS1, there are plenty of previous researches have studied. For example, it has been proven that circPDSS1 is conducive to gastric cancer progression via sequestering miR-186-5p and regulating NEK2 [11]. CircPDSS1 activates Wnt/β-catenin pathway to stimulate the colorectal cancer development [12]. Additionally, circPDSS1 has been ascertained to promote bladder cancer via sequestering miR-16 [13]. Nonetheless, the molecular and regulatory mechanisms of circPDSS1 in BC have not been studied, so the potential role and mechanism of circPDSS1 in BC aroused our interest.

Accordingly, we aimed to explore the molecular mechanism and functions of circPDSS1 in BC cells, which might be helpful for the pathological study on BC.

## Materials and methods

### Cell culture

Human BC cells (MCF-7, MDA-MB-231, SKBR-3, BT-549, and BT-474) and normal human breast epithelial cells (MCF-10A) were acquired from Chinese Academy of Sciences (Beijing, China). Cells were cultured in RPMI-1640 medium (Invitrogen, Carlsbad, CA, USA) blended with 1% penicillin/streptomycin (Sigma-Aldrich, Milan, Italy) and 10% fetal bovine serum (FBS; Invitrogen). Cell plates were kept in an incubator with 5% CO_2_ at 37 °C.

### Cell transfection

MDA-MB-231 and MCF-7 cells were transfected with interference sequences targeting circPDSS1 (sh-circPDSS1#1/#2) or CKAP5 (sh-CKAP5#1/#2) and their corresponding negative controls (sh-NCs). The miR-320c mimics, NC inhibitor and NC mimics were procured from GenePharma (Shanghai, China). MiR-320c inhibitor (Merck, Darmstadt, Germany) was also purchased and its sequence was as follows: accctctcaacccagctttt. As the single-stranded RNA obtained by chemical synthesis and specific modifications, miR-320c inhibitor could specifically bind with mature miRNA to inhibit miRNA function. Cells were transfected into cells with the application of Lipofectamine 2000 (Invitrogen) in the present study.

### Quantitative real-time polymerase chain reaction (qRT-PCR)

First of all, total RNAs were isolated from cells (1.3 × 10^5^) utilizing TRIzol reagent (Invitrogen). Next, RNAs were reversely transcribed into complementray DNAs (cDNAs) employing Reverse Transcription Kit (Applied Biosystems, Foster City, CA, USA). The qRT-PCR detection was conducted in Bio-Rad CFX96 system (Takara, Tokyo, Japan) with SYBR-Green Real-Time PCR Kit (Takara). Fold expression changes were calculated by 2^−ΔΔCt^ method [14]. GAPDH or U6 was used as internal reference for circPDSS1/linear PDSS1/CKAP5 or miRNAs.

### RNase R treatment and actinomycin D (ActD) assays

With the aim of blocking transcription of RNAs (linear PDSS1 and circPDSS1), 2 mg/ml ActD or dimethylsulphoxide (both from Sigma-Aldrich) was added into the culture medium containing 3000–5000 cells. As for RNase R treatment, total RNAs (2 μg) from 2 × 10^6^ cells were treated with 3 U/μg of RNase R (Epicentre Technologies, Madison, WI, USA) for half an hour. In the end, qRT-PCR was performed to examine the expression of circPDSS1 or linear PDSS1.

### cell-counting kit 8 (CCK-8) assay

Cells (1 × 10^3^) were first inoculated on a fresh 96-well plate. At different time points (0, 24, 48, 72, or 96 h), 10 µL CCK-8 reagents were added into these wells. After incubation, the absorbance value of each well was measured at 450 nm via a microplate reader (Bio-Tek Instruments, Hopkinton, MA, USA).

### Colony formation assay

Transfected cells were plated in 6-well plates (500/well) for 14 days. Then, cells were treated with paraformaldehyde (Sigma-Aldrich) and dyed in crystal violet (Sigma-Aldrich). In the end, the quantity of colonies was counted manually.

### Wound healing assay

Briefly, 5 × 10^4^ transfected cells were plated into 6-well plates. The surface of the cell monolayers was scratched by a sterile pipette tip to create a wound when the cell confluence reached 80%. The created wound was immediately photographed. After cells were cultured for another 24 h, wound width was observed with an inverted microscope (Olympus, Tokyo, Japan).

### Terminal-deoxynucleoitidyl transferase mediated nick end labeling (TUNEL) assay

Cell apoptosis was assessed using the in situ cell death detection kit (Boster Bio-tech, Wuhan, China) as instructed. Cells (3600–4200) were first cultured. After cell nuclei were stained with 4′,6-diamidino-2-phenylindole (DAPI; Sigma-Aldrich), cells were observed by a fluorescence microscope (Olympus).

### Transwell-invasion assay

Cell invasion ability was tested by transwell assays with the application of transwell chambers (Corning, NY, USA). Following transfection, BC cells (8000–36,000) were plated into the top compartment with free serum medium, while the medium blended with 10% FBS was put in the lower compartment. Moreover, Matrigel (Millipore, Billerica, MA, USA) was pre-coated on the upper chamber before the plating of cells. After 48-h incubation, cells were fixed by methanol and then dyed with crystal violet. Cells invaded to the bottom chamber were observed via a microscope (Olympus) and 5 fields were randomly chosen under ×100 magnification.

### Immunofluorescence (IF) Assay

Cells (8000–10,000) were fixed with paraformaldehyde and then permeabilized using 0.5% Triton X-100 for 5 min. After phosphate-buffered saline (PBS; Solarbio, Beijing, China) washing, cells were cultured with primary antibodies including anti-N-cadherin, anti-E-cadherin, anti-ZEB1, and anti-Vimentin. Cells were cultivated severally with FITC and TRITC conjugated with secondary antibody for 2 h after PBS rinsing. Later, nuclei were treated with DAPI for counterstain. Cells were observed through a confocal microscope (Olympus) at last.

### Subcellular fractionation assay

Cytoplasmic and Nuclear RNA Purification Kit (Norgen, Ontario, Canada) was utilized to separate and purify cell nuclei and cytoplasm fragments from BC cells (8 × 10^5^). Expression of circPDSS1, U6 (nuclear reference) and GAPDH (cytoplasmic reference) was assessed by qRT-PCR analysis.

### Fluorescence in situ hybridization (FISH) assay

Cellular location of circPDSS1 was determined by the FISH Kit (Roche, Basel, Switzerland). BC cells (8000–10,000) were fixed with paraformaldehyde. Subsequently, digoxigenin-labeled circPDSS1 probes (Sigma-Aldrich) were incubated with the cells. Antagonistic circPDSS1 probes were produced and used as NC. Nuclei were stained by Hoechst (Sigma-Aldrich) for 10 min. Then, the fluorescence images were acquired by a laser confocal scanning microscopy (Olympus).

### Western blot

Total proteins were extracted from BC cells (1 × 10^7^) via radioimmunoprecipitation assay (RIPA) lysis buffer (Invitrogen). bicinchoninic acid (BCA) protein assay kit was used to examine protein concentrations, and then proteins were isolated by 10% sodium dodecyl sulfate–polyacrylamide gel electrophoresis (SDS-PAGE). Next, proteins were shifted to polyvinylidene fluoride (PVDF) membranes which were blocked with 5% defatted milk. After that, primary antibodies for CKAP5 (ab86073, Abcam, Cambridge, USA) and GAPDH (ab8245, Abcam) were put on the membranes for incubation. Subsequently, secondary antibodies were also added to incubate with the membranes. Finally, protein bands were visualized with the application of the chemiluminescence system.

### Luciferase reporter assay

The sequence of circPDSS1, CKAP5 or SRSF7 covering wild-type (WT) or mutant (Mut) binding sites on miR-320c was sub-cloned into pmirGLO vectors to generate circPDSS1-WT/Mut, CKAP5-WT/Mut or SRSF7-WT/Mut. Then the constructed vectors were co-transfected with NC mimics or miR-320c mimics into MDA-MB-231 and MCF-7 cells (1.2 × 10^5^). The luciferase activity was detected by Dual-Luciferase Reporter Assay System (Promega, USA).

### RNA pull down assay

The biotinylated probes of circPDSS1 (Bio-circPDSS1) and Bio-NC were constructed by GenePharma. MDA-MB-231 and MCF-7 cells (1 × 10^7^) were lysed and cell lysates were co-cultured with the M-280 streptavidin magnetic beads (Sigma-Aldrich) and biotinylated probes. Relative enrichment of miRNAs was detected by qRT-PCR.

### RNA binding protein immunoprecipitation (RIP) assay

Based on a former study, specific miRNAs can be recruited to the cytoplasmic RNA induced silencing complex (RISC), and Ago2, a vital component of RISC, promotes the binding of miRNA to target mRNA [15]. RIP assay was conducted to determine the enrichment of circPDSS1, miR-320c and CKAP5 in anti-Ago2, whose results could reflect their binding relationship. EZ-Magna RIP kit (Millipore, Billerica, MA, USA) was applied to perform RIP experiments. MCF-7 and MDA-MB-231 cells (1 × 10^7^) were cultured in RIP lysis buffer. Then, cell lysates were incubated with anti-IgG (Millipore) and anti-Ago2 (Millpore) conjugated with Protein A/G Agarose. In the end, the enrichment of circPDSS1, miR-320c and CKAP5 in the precipitates was evaluated through qRT-PCR.

### Statistical analysis

The data in this research were analyzed via GraphPad Prism 6.0 (La Jolla, CA, USA) and manifested as mean ± standard deviation (SD). Parametric statistics was done via utilizing student’s t-test or one-way/two-way analysis of variance (ANOVA). Above experiments were conducted at least three times. P < 0.05 was set as the threshold for statistical significance.

## Results

### CircPDSS1 knockdown restricts the proliferation, migration, invasion, and EMT of BC cells

**Fig. 1:**
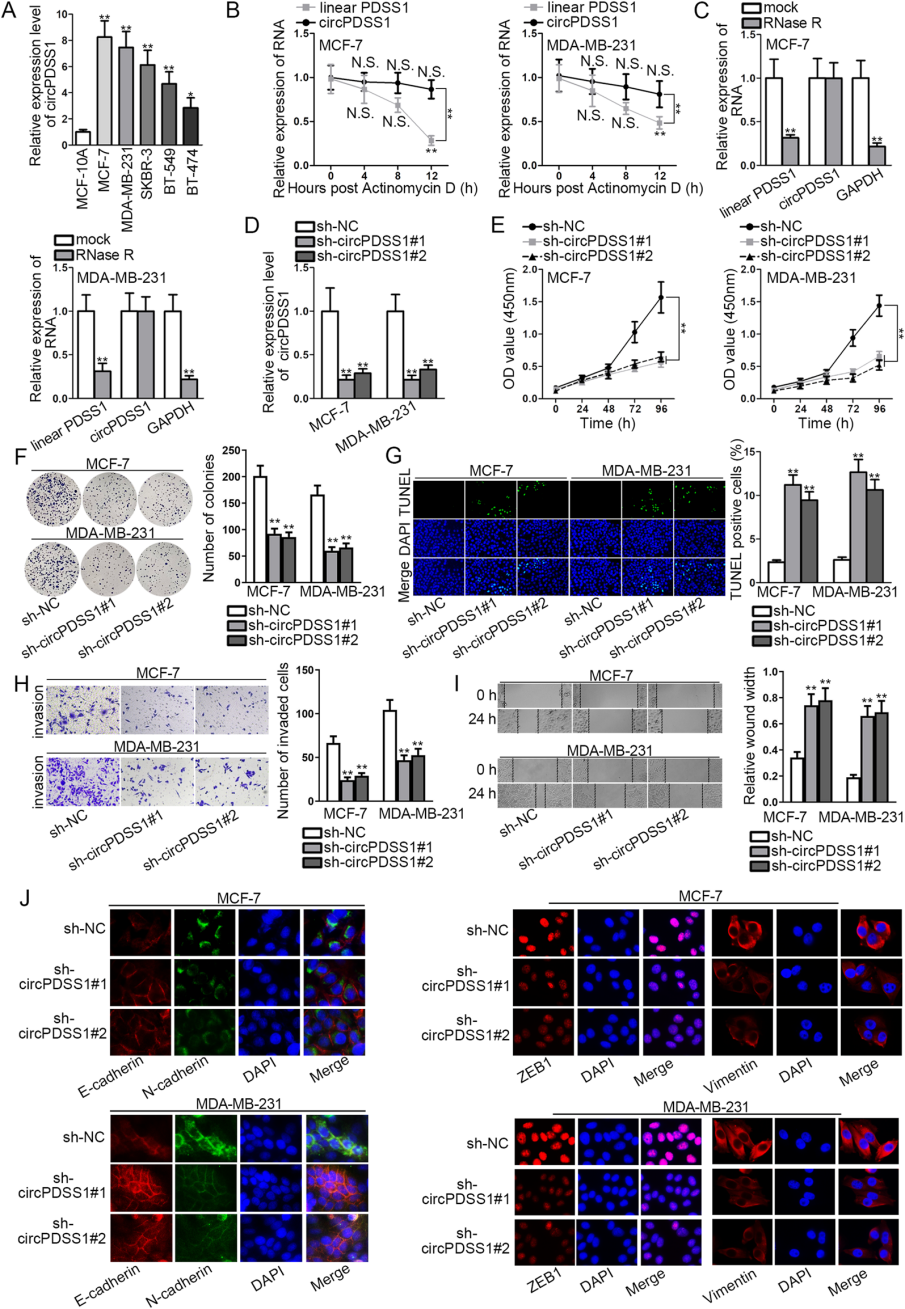
CircPDSS1 promotes the proliferation, migration, invasion and EMT of BC cells. **A** The expression of circPDSS1 in BC cell lines (MCF-7, MDA-MB-231, SKBR-3, BT-549 and BT-474) and normal mammary epithelial cell line (MCF-10A) was measured via qRT-PCR. **B**, **C** The expression of circPDSS1 and linear PDSS1 in ActD or RNase R-treated BC cells was analyzed at the time point 0, 4, 8, and 12 h via qRT-PCR. **D** CircPDSS1 expression in sh-circPDSS1#1/#2-transfected cells was detected by qRT-PCR. **E**, **F** CCK-8 and colony formation assays were employed to examine the proliferation of the transfected BC cells. **G** TUNEL assay was applied to test the apoptosis of BC cells. **H**, **J** Transwell, wound healing and IF assays were utilized to analyze the invasion, migration and EMT of the transfected BC cells. ^*^P < 0.05, ^**^P < 0.01. n.s.: no significance

The cancer-promoting role of circPDSS1 has been validated in gastric cancer [11]. However, the specific role of circPDSS1 in BC remains largely unknown. Thus, we aimed to explore its function in BC cells. Firstly, qRT-PCR analysis suggested that circPDSS1 was up-regulated in BC cell lines (MCF-7, MDA-MB-231, SKBR-3, BT-549, and BT-474), comparing to normal mammary epithelial cells (MCF-10A) (Fig. 1A). As circPDSS1 was expressed highest in MCF-7 (ER-positive BC cell line) and MDA-MB-231 (Triple negative BC cell line) cell lines among the listed BC cells, we involved MDA-MB-231 and MCF-7 cells in the following investigation. After cells were treated with ActD or RNase R, we observed that the expression of circPDSS1 had no remarkable change at different time points and that of linear PDSS1 diminished with time, which confirmed the high stability of circPDSS1 (Fig. 1B, C). Further, the high knockdown efficacy of sh-circPDSS1#1/#2 was testified in BC cells (Fig. 1D). Subsequently, CCK-8 and colony formation assays revealed circPDSS1 depletion hindered BC cell proliferation, which was indicated by the decreased OD value and colony number in treated groups (Fig. 1E, F). Meanwhile, TUNEL assay reflected that circPDSS1 knockdown induced BC cell apoptosis, as the number of TUNEL positive cells was increased after circPDSS1 deficiency (Fig. 1G). Moreover, transwell and wound healing assays manifested that the number of invaded cells was decreased and the wound closure was slower because of circPDSS1 depletion, which represented the invasive and migratory capabilities of circPDSS1-depleted cells were repressed (Fig. 1H, I). Finally, IF assay uncovered that the fluorescence intensity of E-cadherin (epithelial marker) was strengthened, whereas that of N-cadherin, ZEB1 and Vimentin (mesenchymal markers) was decreased by circPDSS1 knockdown in BC cells (Fig. 1J). In brief, circPDSS1 is upregulated in BC cells, and circPDSS1 knockdown represses BC cell proliferation, invasion, migration, and EMT.

### MiR-320c is the downstream gene of circPDSS1 and miR-320c overexpression inhibits BC cell growth

**Fig. 2:**
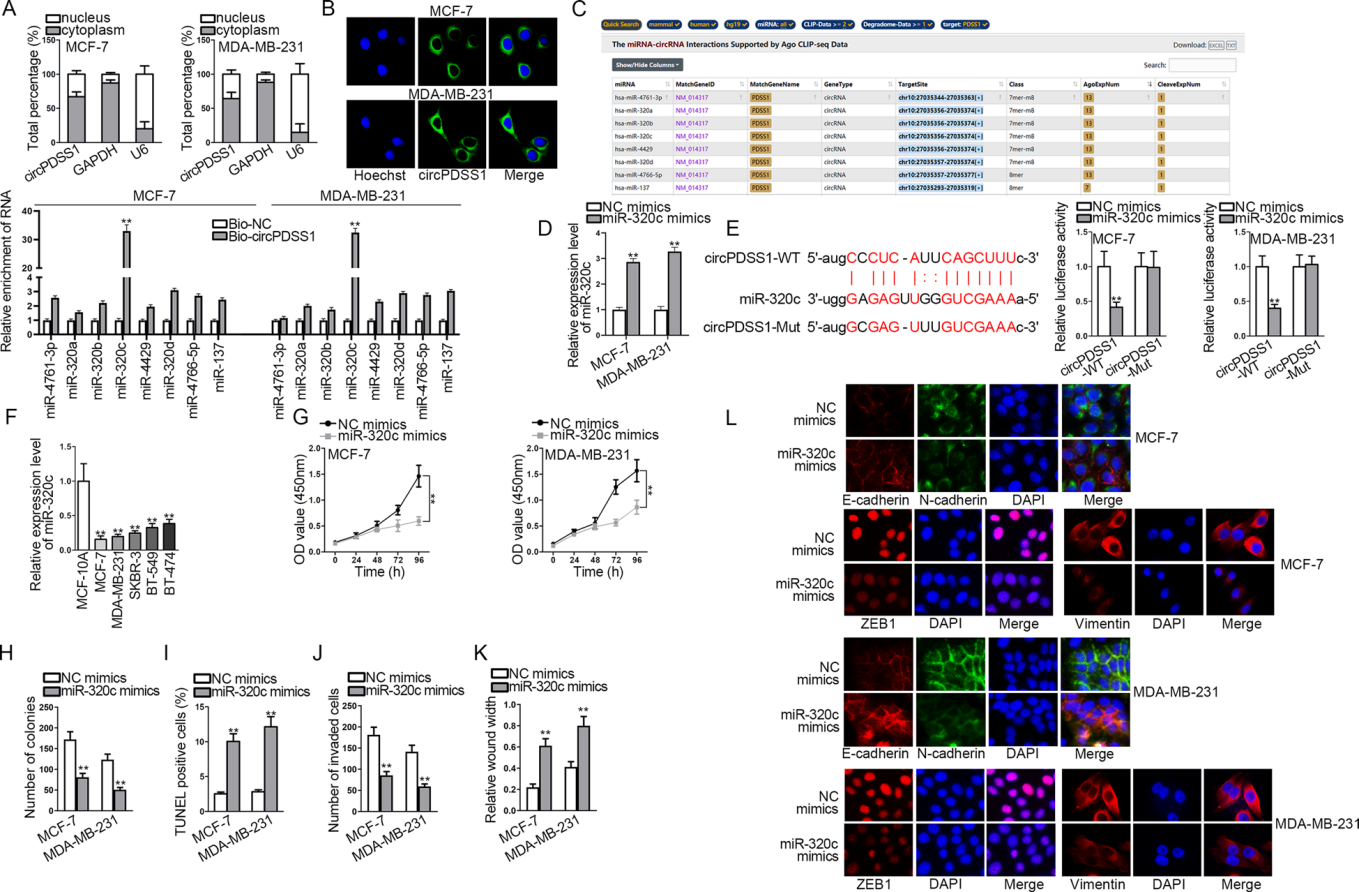
MiR-320c is targeted by circPDSS1 and miR-320c overexpression inhibits BC cell malignant behaviors. **A**, **B** Subcellular fractionation and FISH assays were implemented to determine the distribution of circPDSS1 in the nuclei and cytoplasm of BC cells. **C** RNA pull down assay measured the RNA enrichment of 8 miRNAs screened out through starBase that possibly bind to circPDSS1. **D** The expression of miR-320c in BC cells transfected with miR-320c mimics was analyzed via qRT-PCR. **E** Luciferase reporter assay was applied for confirming the binding sites between circPDSS1 and miR-320c acquired from starBase. **F** MiR-320c expression in BC cells and mammary epithelial cells was measured via qRT-PCR. **G**, **H** CCK-8 and colony formation assays were implemented to measure the proliferation of the transfected BC cells. **I** TUNEL assay was used to test the apoptosis of BC cells upon miR-320c overexpression. **J**, **L** Transwell, wound healing and IF assays were conducted to examine the invasion, migration and EMT of the transfected BC cells. ^**^P < 0.01

First of all, subcellular fractionation and FISH assays were implemented to detect the distribution of circPDSS1 in MCF-7 and MDA-MB-231 cells. Collected data manifested that circPDSS1 was basically distributed in BC cell cytoplasm (Fig. 2A, B). Next, through starBase (http://starbase.sysu.edu.cn/index.php), 8 miRNAs (miR-4761-3p, miR-320a, miR-320b, miR-320c, miR-4429, miR-320d, miR-4766-5p, and miR-137) that possibly bound to circPDSS1 were screened. Therefore, RNA pull down assay was utilized to test the enrichment of the above miRNAs in Bio-circPDSS1 group. We found that miR-320c was the most enriched in Bio-circPDSS1, among all miRNA candidates (Fig. 2C). Hence, miR-320c was determined as the downstream target of circPDSS1. In BC cells, miR-320c expression was upregulated by the transfection of miR-320c mimics (Fig. 2D). Subsequently, the binding sites between circPDSS1 and miR-320c predicted by starBase were shown in Fig. 2E. Then, luciferase reporter assay ensured the effectiveness of the binding sites, for miR-320c mimics obviously weakened the luciferase activity of circPDSS1-WT but had little effect on that of circPDSS1-Mut. Moreover, miR-320c expression in BC cells and mammary epithelial cells was measured by qRT-PCR, and it turned out that miR-320c was downregulated in BC cell lines in comparison with MCF-10A cell line (Fig. 2F). Then, we performed functional experiments to uncover the underlying influence of miR-320c on BC cell phenotype. CCK-8 and colony formation assays revealed that miR-320c overexpression restrained the proliferation of BC cells (Fig. 2G, H). In the meantime, TUNEL assay indicated that BC cell apoptosis process was expedited after miR-320c augment (Fig. 2I). Additionally, transwell and wound healing assays proved that BC cell invasion and migration were suppressed by miR-320c upregulation (Fig. 2J, K). IF assay demonstrated that miR-320c mimics restricted the EMT process of BC cells (Fig. 2L). In short, miR-320c is sponged by circPDSS1, and miR-320c overexpression inhibits malignant phenotype of BC cells.

### CKAP5 is targeted by miR-320c in BC cells

**Fig. 3:**
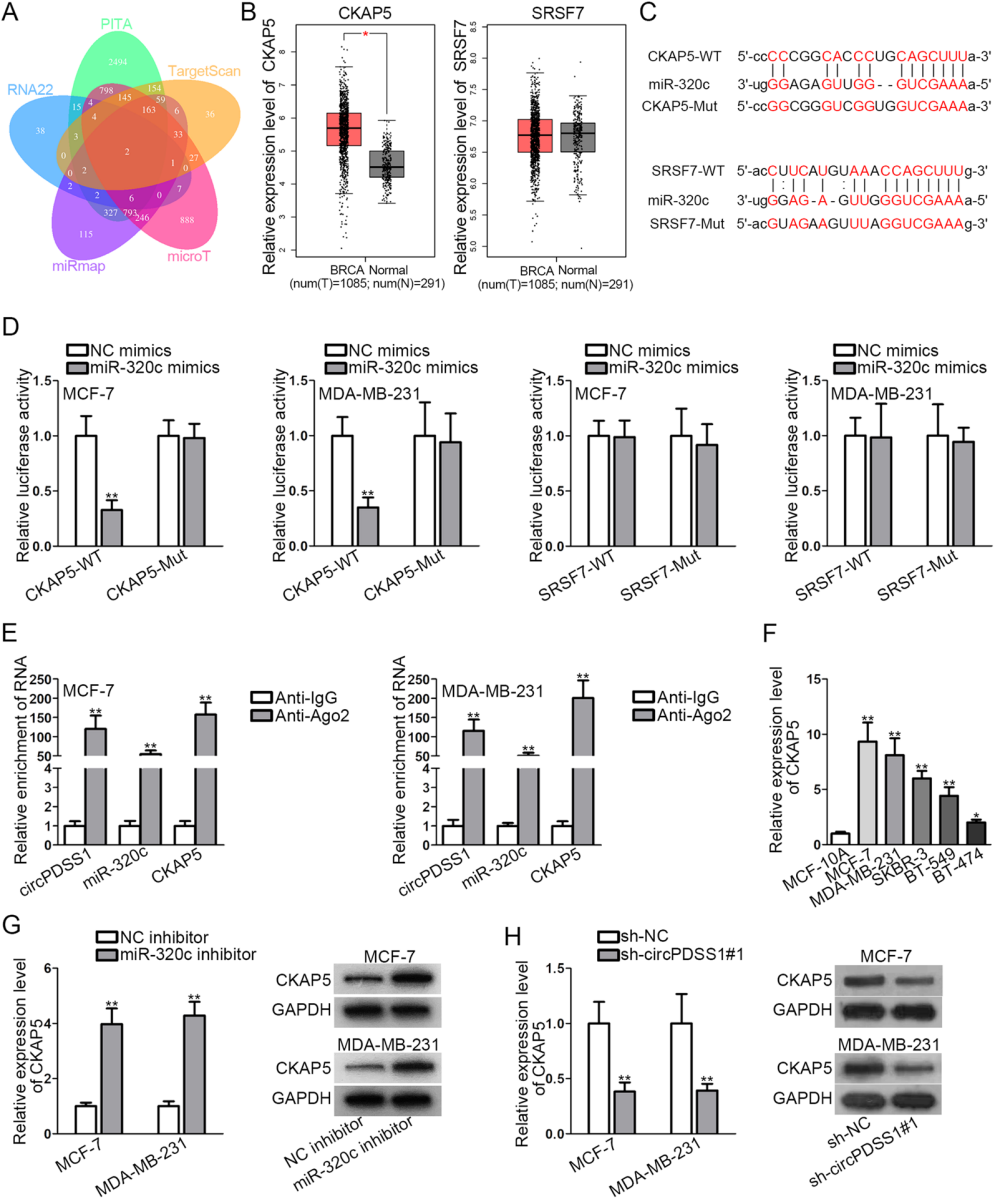
CKAP5 is targeted by miR-320c in BC cells. **A** Two target genes of miR-320c were screened out through PITA, RNA22, miRmap, TargetScan, and microT databases. **B** The expression of CKAP5 and SRSF7 in 1085 BRCA tissues (breast invasive carcinoma) and 291 normal breast tissues was analyzed via GEPIA database. **C** The binding sites between miR-320c and CKAP5/SRSF7 were conjectured by starBase. **D** Luciferase reporter assays tested the luciferase activity of CKAP5/SRSF7-WT and CKAP5/SRSF7-Mut after BC cells were transfected with NC mimics or miR-320c mimics. **E** RIP assays measured the RNA enrichment of circPDSS1, miR-320c and CKAP5 in Anti-IgG or Anti-Ago2 precipitates. **F** The expression of CKAP5 in BC cells and mammary epithelial cells was analyzed via qRT-PCR. **G** The expression of CKAP5 in BC cells was tested by qRT-PCR and western blot assays after miR-320c inhibition. **H** qRT-PCR and western blot assays explored the mRNA and protein levels of CKAP5 in BC cells after circPDSS1 downregulation. ^*^P < 0.05, ^**^P < 0.01

To explore the target gene of miR-320c, 2 mRNAs, namely CKAP5 and SRSF7, were predicted via PITA, RNA22, miRmap, microT, and TargetScan databases (Fig. 3A). Moreover, based on the public database, GEPIA (http://gepia.cancer-pku.cn/), CKAP5 was up-regulated in BRCA (breast invasive carcinoma) tissues relative to normal breast tissues, whereas SRSF7 was not differentially expressed in BRCA tissues and normal tissues (Fig. 3B). To investigate the interaction between miR-320c and CKAP5/SRSF7, following assays were conducted. First, the binding sites of miR-320c with CKAP5 or SRSF7 were predicted according to starBase (Fig. 3C). Then, data from luciferase reporter assays revealed that the luciferase activity of CKAP5-WT was repressed by upregulated miR-320c while that of SRSF7-WT was not obviously affected (Fig. 3D). This finding implied that CKAP5 was directly targeted by miR-320c in BC cells. Thereby, CKAP5 was chosen for the following assays. Next, we performed RIP assay finding that circPDSS1, miR-320c and CKAP5 co-existed in RISC in BC cells (Fig. 3E), which supported the possibility of ceRNA network [16]. In addition, it was verified that CKAP5 was upregulated in BC cells (Fig. 3F). Based on qRT-PCR results, the mRNA and protein levels of CKAP5 were increased in response to miR-320c inhibition (Fig. 3G). Moreover, the knockdown of circPDSS1 reduced the mRNA and protein levels of CKAP5 (Fig. 3H). To sum up, CKAP5 is targeted by miR-320c, and CKAP5 expression is negatively modulated by miR-320c while being positively regulated by circPDSS1 in BC cells.

### CircPDSS1 promotes BC cell growth via regulating miR-320c/CKAP5 axis

**Fig. 4:**
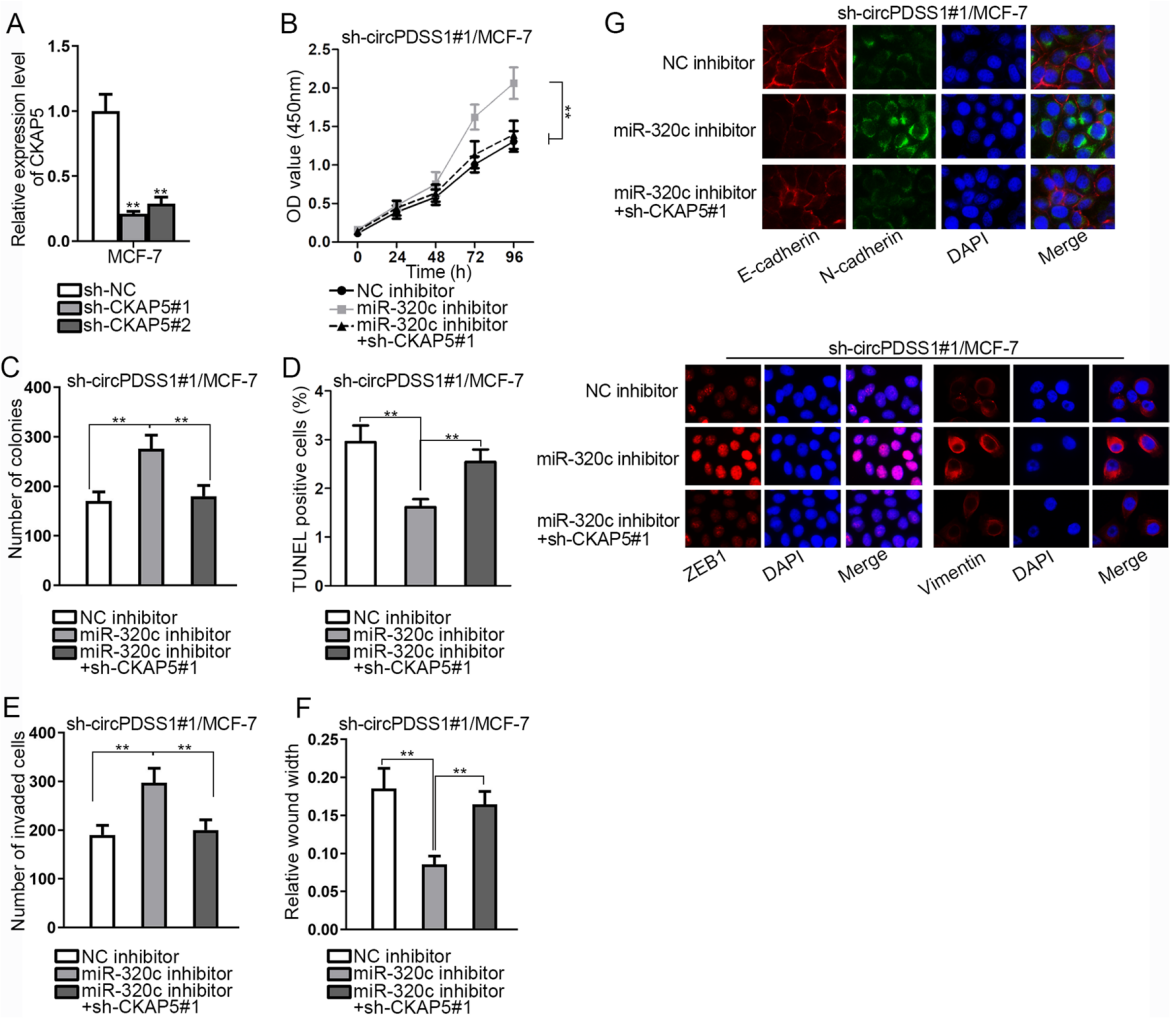
CircPDSS1 accelerates BC cell growth via regulating CKAP5/miR-320c axis. **A** qRT-PCR measured CKAP5 expression after the transfection of sh-CKAP5. **B**, **C** BC cell proliferation was analyzed by CCK-8 and colony formation assays. **D** TUNEL assay was utilized to test the apoptosis of the transfected BC cells. **E**–**G** Transwell, wound healing and IF assays were implemented to analyze the invasion, migration and EMT of the transfected BC cells. ^**^P < 0.01

To test whether circPDSS1 could affect the phenotype of BC cells through targeting miR-320c/CKAP5 axis, rescue experiments were carried out. At the beginning, the high efficacy of CKAP5 knockdown was validated in BC cells (Fig. 4A). Subsequently, CCK-8, colony formation and TUNEL assays indicated that CKAP5 depletion respectively abrogated the stimulating or suppressive effect of miR-320c inhibition on the proliferation or apoptosis of circPDSS1-depleted BC cells (Fig. 4B–D). Transwell and wound healing assays manifested that CKAP5 silencing counteracted the promoting influence of miR-320c inhibition on the invasion and migration of circPDSS1-reduced BC cells (Fig. 4E, F). Similarly, IF assay demonstrated that CKAP5 downregulation offset the facilitating influence of miR-320c inhibition on the EMT of circPDSS1-depleted BC cells (Fig. 4G). In conclusion, circPDSS1 facilitates BC cell growth via regulating miR-320c/CKAP5 axis.

## Discussion

BC is threatening women’s health around the world. Referring to the public data, BC is the second main cause of cancer-linked deaths [17, 18]. The chosen treatment methods for BC are based on the stage of cancer and individual general condition, as well as personal preferences. Although significant progress has been achieved in diagnosing and treating BC, the five-year overall survival rate is still not favorable [19]. The etiopathogenesis of BC is associated with the aberrant expression of numerous tumor-promoter or suppressor genes. Accordingly, revealing the underlying mechanisms of BC oncogenesis and development is indispensable for identifying available diagnosis markers and effective treatment strategies for BC patients.

Recently, increasing evidence has shown that circRNAs can be used as biomarkers to predict and analyze the clinical severity of cancers, such as cholangiocarcinoma [20], osteosarcoma [21], lung adenocarcinoma [22], pancreatic ductal adenocarcinoma [23], as well as BC [24]. The regulatory effects of some circRNAs have also been investigated. For example, circRNA-0001982 prompts BC cell proliferation but restrains cell apoptosis via targeting miR-143 [25]. CircPDSS1 is a novel circRNA in cancer research and has been reported to facilitate gastric cancer development via binding with miR-186-5p and modulating NEK2 [11]. However, the role of circPDSS1 in BC has not been well studied, for which we aimed to reveal the potential function of circPDSS1 in BC. In this research, we verified the high expression of circPDSS1 in BC cells. Moreover, it was found that depletion of circPDSS1 hampered BC cell proliferation, migration, invasion as well as EMT, suggesting that circPDSS1 served as an oncogene in BC cells.

Growing literature has demonstrated that a variety of RNAs including long non-coding RNAs (lncRNAs) and circRNAs could act as ceRNAs via competitively binding with miRNAs to upregulate the downstream gene expression post-transcriptionally in cancer development and progression [26, 27]. For instance, circRHOT1 prompts malignant progression of BC through regulating miR-106a-5p/STAT3 axis [28]. CircFOXK2 contributes to the development of BC by upregulating IGF2BP3 expression and sequestrating miR-370 [29]. In our research, circPDSS1 was ascertained to bind to miR-320c. The tumor-suppressor role of miR-320c has been verified in cancers. For example, miR-320c targets CDK6 to inhibit bladder cancer [30]. MiR-320 family members, including miR-320c, are low expressed in colorectal adenoma and influences cell proliferation through targeting CDK6 [31]. MiR-320c acts as a potential tumor-inhibitor gene that is epigenetically suppressed by PRC2 in multiple myeloma [32]. It has been found that miR-320c maintains chemosensitivity of BC to make chemotherapy for BC efficient [33]. In this study, miR-320c was observed to be low-expressed in BC cells. In addition, miR-320c overexpression impeded BC cell malignant processes, including cell proliferation, invasion, migration, as well as EMT. Further, our research verified that CKAP5 was targeted by miR-320c in BC cells. Previous literature has uncovered the CKAP5 is linked to the poor prognosis of non-small cell lung cancer patients [34]. CKAP5 has been pointed out to be a potential prognostic and key gene in HCC according to Kaplan–Meier survival analysis [35]. Moreover, CKAP5 has been confirmed to be crucial for glioma cell migration and proliferation [36]. In this current study, CKAP5 expression was discovered to be obviously upregulated in BC cells. In addition, the expression of CKAP5 was negatively modulated by miR-320c, but had a positive interaction with circPDSS1. Furthermore, CKAP5 downregulation abrogated the promoting influence of miR-320c depletion on BC cell proliferation, migration, invasion, and EMT.

This study mainly concentrated on triple-negative BC and ER-positive BC, which were represented by MDA-MB-231 and MCF-7 cells respectively. We only explored the mechanism of circPDSS1/miR-320c/CKAP5 axis in BC cells whereas other possible mechanisms and pathways were not investigated, such as the potential combination between miR-320c and CDK6 in BC cells.

## Conclusion

This study uncovered that CircPDSS1 facilitates BC cell proliferation, invasion, migration and EMT via modulating miR-320c/CKAP5 axis, which might provide some novel insights for researching BC pathology.

## Data Availability

Research data are not shared.

## Abbreviations

ActD: Actinomycin D
ANOVA: Analysis of variance
BC: Breast cancer
BCA: Bicinchoninic acid
Bio: Biotinylated
CCK-8: Cell-counting kit 8
CDNAs: Complementray DNAs
CeRNA: Competitive endogenous RNA
CircRNAs: Circular RNAs
DAPI: 4′,6-Diamidino-2-phenylindole
EdU: 5-ethynyl-20-deoxyuridine
EMT: Epithelial-mesenchymal transition
FBS: Fetal bovine serum
FISH: Fluorescence in Situ Hybridization
IF: Immunofluorescence
MiRNA: microRNA
MRNA: Messenger RNA
Mut: Mutant
PBS: Phosphate-buffered saline
PVDF: Polyvinylidene fluoride
QRT-PCR: Quantitative real-time polymerase chain reaction
RIP: RNA Binding Immunoprecipitation
RIPA: Radioimmunoprecipitation assay
RISC: RNA induced silencing complex
SD: Standard deviation
SDS-PAGE: Sodium dodecyl sulfate–polyacrylamide gel electrophoresis
Sh-NCs: Negative controls
TCGA: The Cancer Genome Atlas
TUNEL: Terminal-deoxynucleoitidyl Transferase Mediated Nick End Labeling
WT: Wild-type
